# The 3.4 GHz BAW RF Filter Based on Single Crystal AlN Resonator for 5G Application

**DOI:** 10.3390/nano12173082

**Published:** 2022-09-05

**Authors:** Rui Ding, Weipeng Xuan, Shurong Dong, Biao Zhang, Feng Gao, Gang Liu, Zichao Zhang, Hao Jin, Jikui Luo

**Affiliations:** 1 Key Lab of Advanced Micro/Nano Electronic Devices & Smart Systems of Zhejiang, College of Information Science and Electronic Engineering, Zhejiang University, Hangzhou 310063, China; 2 MOE Frontier Science Center for Brain Science & Brain-Machine Integration, Zhejiang University, Hangzhou 310063, China; 3 ZJU-Hangzhou Global Scientific and Technological Innovation Center, Hangzhou 311200, China; 4 Ministry of Education Key Lab of RF Circuits and Systems, College of Electronics & Information, Hangzhou Dianzi University, Hangzhou 310061, China; 5 Innovation and Research Institute of HIWING Technology Academy, Beijing 100074, China

**Keywords:** film bulk acoustic resonator, bandpass filter, single crystal AlN, 5G communication

## Abstract

To meet the stringent requirements of 5G communication, we proposed a high-performance bulk acoustic wave (BAW) filter based on single crystal AlN piezoelectric films on a SiC substrate. The fabrication of the BAW filter is compatible with the GaN high electron mobility transistor (HEMT) process, enabling the implementation of the integration of the BAW device and high-performance monolithic microwave integrated circuit (MMIC). The single crystal AlN piezoelectric film with 650-nm thickness was epitaxially grown on the SiC substrate by Metal Organic Chemical Vapor Deposition (MOCVD). After wafer bonding and substrate removal, the single crystal AlN film with electrode layers was transferred to another SiC wafer to form an air gap type BAW. Testing results showed that the fabricated resonators have a maximum Q-factor up to 837 at 3.3 GHz resonant frequency and electromechanical coupling coefficient up to 7.2%. Ladder-type filters were developed to verify the capabilities of the BAW and process, which has a center frequency of 3.38 GHz with 160 MHz 3 dB bandwidth. The filter achieved a minimum 1.5 dB insertion loss and more than 31 dB out-of-band rejection. The high performance of the filters is attributed to the high crystallinity and low defects of epitaxial single crystal AlN films.

## 1. Introduction

Bulk acoustic wave (BAW) filters are widely used in modern mobile communication due to their high Q factor, low insertion loss, and small size [1,2,3]. Meanwhile, the evolution of 5G mobile communication demands the filters to have a higher frequency, larger bandwidth, and lower insertion loss. Compared with polycrystalline AlN thin films that have been widely used in fabricating traditional BAW devices, single crystal AlN thin film has a higher electromechanical coupling coefficient and lower crystal defects, which would lead to larger filter bandwidth and lower insertion loss at high operation frequency. It is thus a promising material for the fabrication of high-performance BAW filters suitable for 5G communication [4,5,6,7]. With the development of GaN active circuits, 5G communication demands compatible processing between BAW filter and GaN HEMT circuits, so that they could be fabricated on single substrates as a monolithic microwave integrated circuit (MMIC) [8]. However, the current AlN BAW process is not compatible with that for GaN circuits, hindering their integration on the same substrate. In this work, a single crystal AlN thin films-based BAW resonator and filter are developed on SiC substrates with a GaN HEMT compatible process, opening an avenue for the integration of filters and active circuits on the same substrate.

## 2. Fabrication of BAW Device

**Figure 1:**
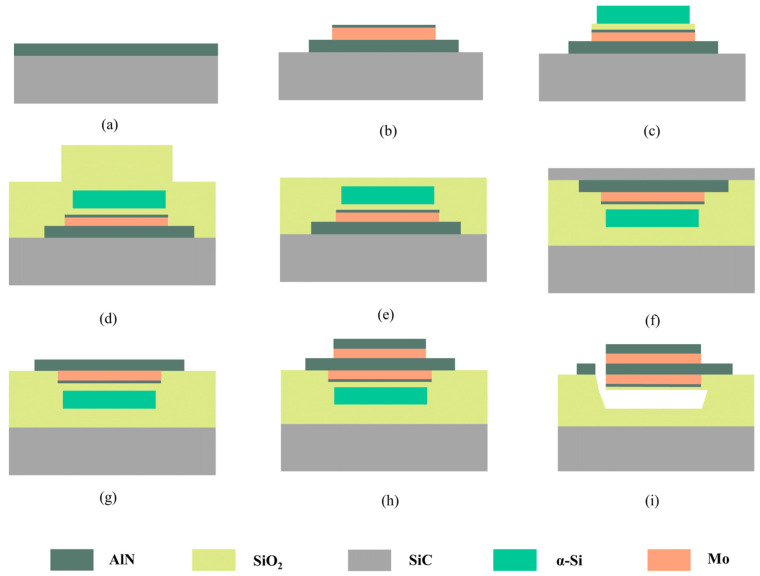
The fabrication process of the single crystal AlN-based BAW filter. (**a**) Deposit single crystal AlN piezoelectric film, (**b**) form the bottom Mo electrode and AlN passive layer by photolithography and etching, (**c**) deposit SiO_2_ and sacrificial α-Si layers in sequence, (**d**) deposit 5-μm SiO_2_ to form the bonding layer, (**e**) remove the surface steps by CMP, (**f**) bond SiO_2_ surface of the wafer to another SiC wafer, (**g**) thin SiC by CMP and etch it with SF6, (**h**) form the top electrode and AlN passive layer by photolithography and etching, (**i**) etch vias to α-Si and release the BAW by removing the sacrificial layer underneath. Note that the dimensions in the drawings are scaled for simplicity.

**Figure 2:**
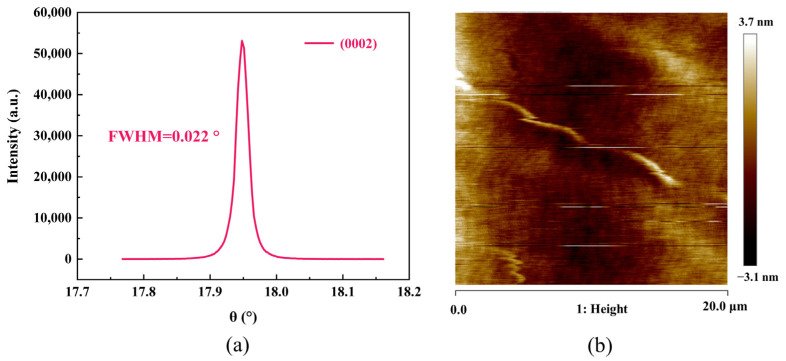
(**a**) XRD spectrum from the Single Crystal AlN piezoelectric film grown by MOCVD, (**b**) surface AFM image of the AlN film.

The fabrication process of the single crystal AlN BAW resonator and filter are shown in Figure 1. Firstly, the 650 nm single crystal AlN piezoelectric film was epitaxially grown on a 4-inch 4H SiC wafer (named top SiC) by Metal Organic Chemical Vapor Deposition (MOCVD) (Figure 1a). The single crystal AlN film has excellent crystallinity, with the orientation of (0002) with a full width half maximum (FWHM) down to 0.022° characterized by X-ray Diffraction (XRD) rocking curve (Figure 2a). Figure 2b shows a 20 × 20 μm AFM scan of the AlN film with an RMS surface roughness of 1.08 nm.

After the deposition of the single crystal AlN, the bottom electrodes were patterned on AlN by sputtering deposition, photolithography, and etching. Baw resonators will excite Lamb waves inevitably when working, which could cause spurious modes and small peaks in the BAW’s impedance response. The geometrical shape of the electrode should not have edges parallel with each other, thus avoiding reflection of Lamb wave between opposite sides. To attenuate the size of the lateral vibration pattern, the shape of the electrode was pentagon in this work, which could effectively prolong the path length of the Lamb wave propagation and minimize the coupling effect between bulk wave resonance and unwanted lateral modes [9]. Molybdenum was selected as the electrode material because of its high acoustic impedance and low acoustic loss. It is worth noting that there is 30-nm AlN deposited on Mo as the passivation layer (Figure 1b). Then the 100-nm SiO_2_ and 1200-nm amorphous silicon (α-Si) layers were deposited on the bottom electrodes. The α-Si serves as a sacrificial layer to form the cavity, while the SiO_2_ here is a protection and support layer (Figure 1c). Subsequently, a 5-μm SiO_2_ layer was deposited to form a bonding layer. The surface was then flattened and polished by Chemical Mechanical Polishing (CMP) (Figure 1d,e).

**Figure 3:**
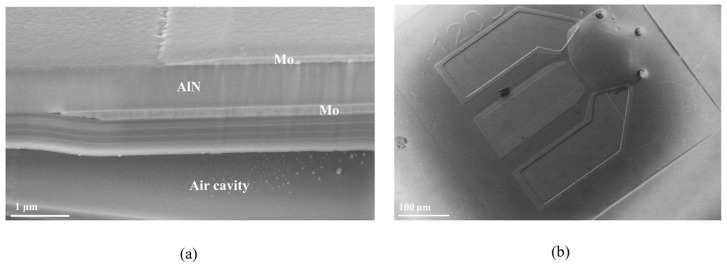
Macro photograph of fabricated device, (**a**) Cross-sectional view of the active area in the resonator, (**b**) SEM image of the resonator.

In the following stage, the above structure was flipped and bonded to another SiC (named bottom SiC) wafer with 1-μm SiO_2_ film on its surface (Figure 1f). The top SiC was thinned to 30 μm and polished by CMP. Next, the AlN piezoelectric layer was exposed by etching SiC with SF_6_ (Figure 1g). After the etching of SiC film, the CMP process was conducted to obtain a smooth AlN surface. Then, the top electrode (Mo) and the corresponding AlN passivation layer were formed by photolithography and etching (Figure 1h). Finally, the through holes to α-Si were etched, and the sacrificial layer α-Si is released by XeF_2_ (Figure 1i). The Scattering parameters (S parameters) of the fabricated resonators and filters were tested with a network analyzer (E5071C, Keysight, Santa Rosa, CA, USA) on a Cascade RF probe station. Figure 3 shows a cross-sectional view and SEM image of the fabricated device. Need to mention that single crystal AlN and GaN film can be both deposited on a single SiC substrate by MOCVD, with GaN HEMT and AlN BAW interconnected each other with metal to form MMIC. Therefore, the whole AlN BAW process is compatible with that for GaN HEMT circuits.

## 3. Results and Discussion

### 3.1. BAW Resonators

**Figure 4:**
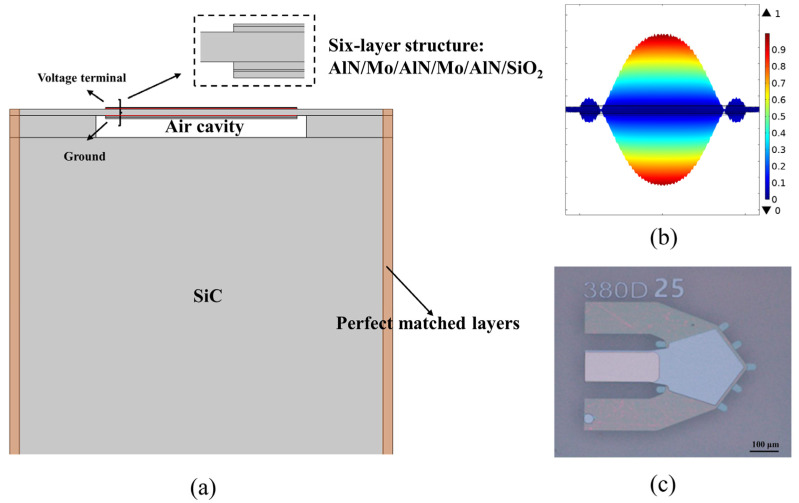
Single crystal AlN BAW resonators, (**a**) Model structure in FEM simulation, (**b**) FEM simulation displacement at the resonant frequency and (**c**) fabricated device.

Multiphysics finite element method (FEM) was used to optimize single crystal AlN BAW resonators. A simplified 2D model of the BAW resonator consisting of the same 6-layer stack as Figure 1i was built for the FEM simulation (Figure 4a). Perfect matched layers surrounding the model were set to suppress wave reflection. By applying RF signals at the top and bottom electrodes, longitudinal acoustic standing waves can be excited within the piezoelectric film as shown in Figure 4b. The fabricated single crystal AlN BAW resonator is shown in Figure 4c.

**Figure 5:**
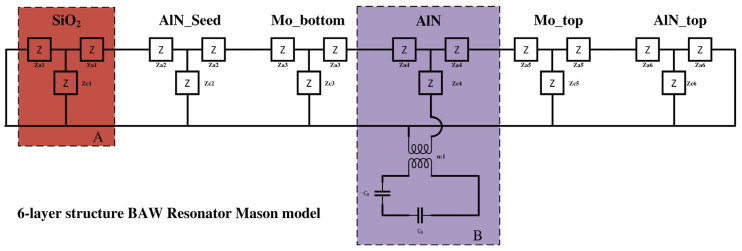
Structure of the Mason model for fabricated BAW resonator. (**A**) represents common acoustic layer and (**B**) represents piezoelectric layer.

Mason model of the BAW device was built for circuit-level design and simulation of the filters (Figure 5). This one-dimensional model obtained the BAW resonator’s input and output impedance characteristics from the equivalent mathematical deformation of the structural and material parameters [10]. Common Acoustic layers were assumed to have only longitudinal sound waves propagating on the *z*-axis and the transmission line model in electromagnetism characterized the transmission of acoustic waves, as shown in Figure 5A. Based on common acoustic layers, the transformer was added to the circuit as a conversion device between mechanical and electrical energy, forming a piezoelectric layer in Figure 5B. At last, the analytical circuit model of BAW was formed by cascading all involved layers.

**Figure 6:**
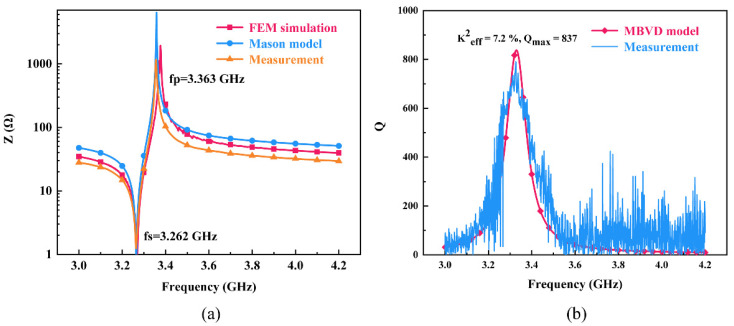
(**a**) Simulated impendence of resonator, (**b**) Q factor of MBVD fit model and measured data.

The impedance characteristics of resonator obtained by FEM and Mason model are shown in Figure 6a. The measured serial resonance frequency (*f_s_*) and parallel resonance frequency (*f_p_*) are 3.262 and 3.363 GHz respectively, which is in good agreement with the simulation results. The effective electromechanical coupling coefficient ($k_{eff}^{2}$) of the resonator is about 7.2%, which was calculated by Equation (1).

(1) $$k_{eff}^{2}=\frac{\pi^{2}}{4}\cdot \frac{f_{s}\cdot \left(f_{p}-f_{s}\right)}{f_{p}^{2}}$$

The $k_{eff}^{2}$ is higher than those of about 6% of traditional polycrystal piezoelectric AlN film-based BAWs resulting from the excellent *c*-axis crystal orientation of the single crystal AlN piezoelectric film. Furthermore, Shealy et al. demonstrated that single crystal AlN-based devices have more than double $k_{eff}^{2}$ than those of polycrystal piezoelectric AlN film-based BAWs because of its better piezoelectric coupling coefficient (*e*_33_) [11], as shown in Equation (2), where *c*_33_ represents longitudinal elastic modulus and *ε_r_* is the relative dielectric constant.

(2) $$k_{t}^{2}=\frac{e_{33}^{2}}{c_{33}\cdot \varepsilon_{r}+e_{33}^{2}}$$

The Q factor is evaluated by method [12] based on the following equation

(3) $$Q=\frac{\omega \cdot \left|S11\right|\cdot group_{delay\left(S11\right)}}{1-{\left|S11\right|}^{2}}$$

Based on the work of Dicke and Bode, the method assumes that resonators are lossless and the Q factors of the circuit vary slowly with respect to frequency. Compared with some traditional Q formulations mentioned in [13], this method will not yield erroneous Q values near *f_s_* and *f_p_* and could provide reliable Q values over a wide range of frequencies. As shown in Figure 6b, the Q factors of the measured data and modified Butterworth Van Dyke (MBVD) model are well fitted with a maximum *Q_max_* of ~837. The figure of merit (FOM) of the fabricated resonator is ~60, calculated by $k_{eff}^{2}$ × *Q_max_*.

### 3.2. BAW Filters

**Figure 7:**
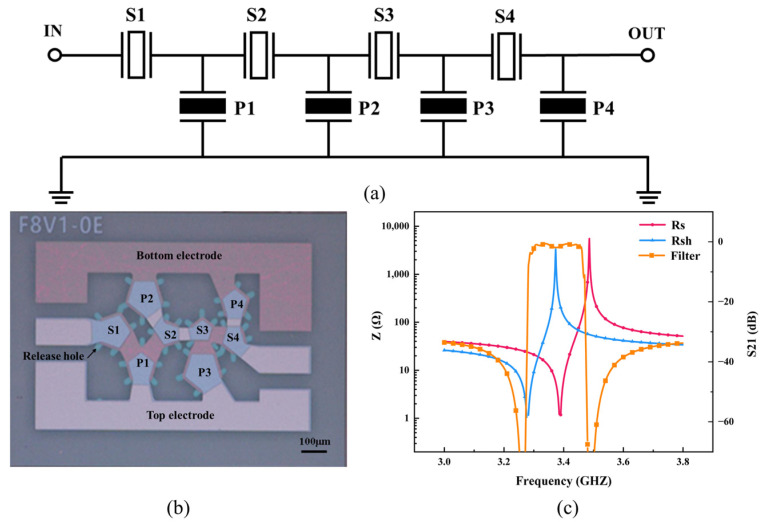
Single crystal AlN BAW filter, (**a**) structure of BAW filter, (**b**) fabricated single crystal BAW filter, and (**c**) simulated results of the series resonator, shunt resonator, and filter.

The filter was designed based on the aforementioned Mason model of the BAW resonators. The higher stage of the filter means higher out-of-band rejection, but it will also increase the total space taken up by designed filters. To get a steep rejection near the passband (about −30 dB) and achieve a good tradeoff between out-of-band rejection and the area of the filter (about 0.65 mm^2^), a 4-stage ladder network filter was designed with a symmetrical layout as shown in Figure 7a. To form a bandpass filter, the *f_p_* of shunt resonators should be set close to *f_s_* of series resonators, achieved by depositing different thicknesses of top Mo electrode on series and shunt resonators. In this work, all series resonators shared the same resonant frequency, as do shunt resonators. On the other side, each resonator had its own distinct area for adjusting passband insertion loss and out-of-band rejection, and smoothed transmission curves to reduce ripples and parasitism. Manual tuning and automatic optimization algorithms like gradient were used to design the top Mo electrode thickness and area of each resonator. The simulated and measured *f_s_* and *f_p_* of the shunt and series resonators are summarized in Table 1. Fabricated single crystal AlN BAW filter is shown in Figure 7b. The size of the filter we fabricated is 1 × 0.65 mm^2^, or 0.6 × 0.35 mm^2^ without a surrounding probe pad, which means it is suitable for miniaturized and integrated packaging.

**Figure 8:**
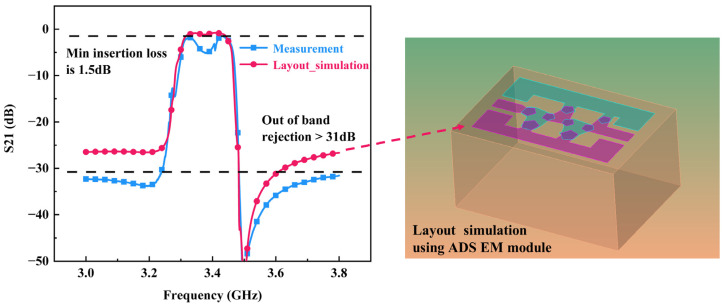
Comparison of the S21 spectra of the filter obtained by layout simulation and measurement.

Figure 7c and Figure 8 show the comparison of filter S21 spectra between the simulation and measurement results. It shows that the filter possesses a center frequency of 3.38 GHz and a 3 dB bandwidth of 160 MHz, fitting well with the simulation results. The minimum insertion loss is 1.5 dB and out-of-band rejection is in excess of 31 dB. Compared with prior work summarized in Table 2, our fabricated resonators have higher $k_{eff}^{2}$ and filters hold smaller insertion loss and size.

From the measured data, there is a transmission dip around the center frequency, resulting from excessive separation between *f_s_* of series resonators and *f_p_* of shunt resonators. As shown in Table 1, the frequency gap between *f_p_* of the shunt resonators and *f_s_* of the series resonators is 50 MHz in the measured filter, which is about 36 MHz larger than that of the simulated data. Because the top Mo electrode thickness of shunt resonators is higher than that of series resonators, we first deposited the top Mo electrode on both series and shunt resonators. Then we deposit the remaining Mo on the shunt resonators and the inaccuracy thickness of the deposited Mo here caused the excessive separation. So, in the fabricating process, accurate controlling of the thickness of the deposited layer to nanometers is needed. After testing, some methods can be performed to eliminate the dip by frequency tuning, such as reducing the resonant frequency of the series resonator by depositing a thin layer of SiO_2_ on its top electrode or trimming the thickness of the top passivation AlN layer to increase the resonant frequency of shunt resonator.

Besides, compared with the Mason model schematic simulation, the left transmission zero (TZ) disappears in the on-chip testing. Simulation with FEM or Mason model defaults that the electric field in FBAR is static, so it is hard to analyze the influence of electromagnetic field distribution and electric field coupling between connecting pads, and substrate. To investigate the problem, we modeled the filter using electromagnetic simulation in an advanced design system (ADS). The result in Figure 8 reveals similar disappearance of left transmission zero, which means different current flow into upper and lower ground lines and effects of connecting pads between resonators can cause this phenomenon.

**Figure 9:**
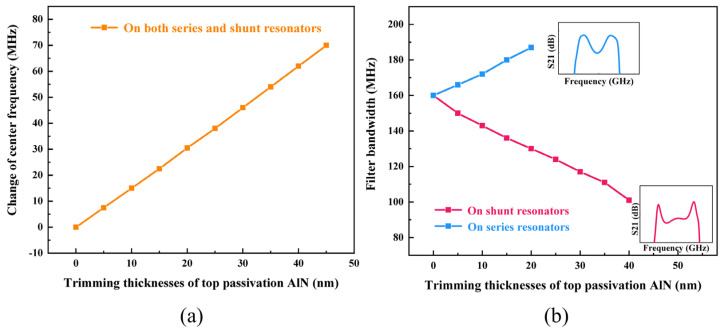
(**a**) Simulated relationship between the change of center frequency and trimming thicknesses of top passivation AlN on both series and shunt resonators; (**b**) simulated relationship between filter 3-dB bandwidth and trimming thicknesses of top passivation AlN on series or shunt resonators.

Sometimes the bandwidth and center frequency of fabricated filters have offset from the expected value, and a method to tune them is needed. Figure 9a shows the simulated increase of center frequency by trimming thicknesses of top passivation AlN on both series and shunt resonators, with a linear relationship of 1.58 MHz/nm. Since we deposited 50 nm passivation AlN on top Mo electrode, the center frequency of the filter has a variable range up to 70 MHz. On the other hand, the bandwidth of the filter is mainly decided by the electromechanical coupling coefficient of the resonator, but it also can be fine-tuned by trimming the top passivation AlN layer. The trimming of the AlN passivation layer on the series resonators will increase the bandwidth while the trimming of that on the shunt resonators will decrease the bandwidth. Because the frequency deviation between series and shunt resonator is already decided when designing originally, the over-tuning of bandwidth will affect the passband performance of the filter, as shown in Figure 9b.

## 4. Conclusions

In summary, a novel single crystal AlN BAW process based on SiC substrate compatible with that for the fabrication of GaN circuits was developed. The resonators have $k_{eff}^{2}$ up to 7.2% and Q factor up to 837. The fabricated BAW filters have a center frequency of 3.38 GHz and a 3-dB bandwidth of 160 MHz, an insertion loss of 1.5 dB, and out of band rejection in excess of 31 dB. The high performance of the filters demonstrates the potential of the single crystal AlN-based BAW technology, which could be broadly used in 5G and future 6G wireless communications.

## Figures and Tables

**Table 1:** Simulated and measured resonant frequency of resonator.

	Shunt Resonator		Series Resonator	
	*f_s_*/GHz	*f_p_*/GHz	*f_s_*/GHz	*f_p_*/GHz
**Simulated**	3.279	3.374	3.388	3.486
**Measured**	3.265	3.356	3.406	3.498

**Table 2:** Comparison with prior BAW resonators and related filters.

Ref.	Freq.	Piezoelectric Layer	$k_{eff}^{2}$ (Resonator)	Q (Resonator)	Min. Insertion Loss (Filter)	Size (Filter)
**[7]**	3.8 GHz	AlN	5.87%	1572	2.01 dB	1.25 × 0.9 mm^2^
**[14]**	2.1 GHz	AlN	6.50%	900	2.15 dB	1.2 × 0.85 mm^2^
**[15]**	2.3 GHz	AlGaN	4.44%	1277	—	—
**[16]**	5.2 GHz	AlN	6.07%	1497	2.00 dB	~1 × 0.7 mm^2^
**this work**	3.4 GHz	AlN	7.20%	837	1.60 dB	0.6 × 0.35 mm^2^

## Data Availability

The data that support the findings of this study are available from the corresponding author upon reasonable request.
